# Exploring the feasibility of patient safety huddles in general practice

**DOI:** 10.1017/S1463423620000298

**Published:** 2020-07-27

**Authors:** Hannah Panayiotou, Charlotte Higgs, Robbie Foy

**Affiliations:** Leeds Institute of Health Sciences, Faculty of Medicine and Health, University of Leeds, Leeds, UK

**Keywords:** general practice, patient safety huddle, survey

## Abstract

**Background:**

Patient safety is a key priority for healthcare systems. Patient safety huddles have been advocated as a way to improve safety. We explored the feasibility of huddles in general practice.

**Methods:**

We invited all general practices in West Yorkshire to complete an online survey and interviewed practice staff.

**Results:**

Thirty-four out of 306 practices (11.1%) responded to our survey. Of these, 22 practices (64.7%) reported having breaks for staff to meet and eight (23.5%) reported no longer having breaks in their practices. Seven interviewees identified several barriers to safety huddles including time and current culture; individuals felt meetings or breaks would not be easily integrated into current primary care structure.

**Discussion:**

Despite their initial promise, there are major challenges to introducing patient safety huddles within the current context of UK general practice. General practice staff may need more convincing of potential benefits.

## Introduction

Patient safety is a key priority for healthcare providers. However, it is estimated that up to 600 incidents occur daily in UK primary care (Esmail, 2013). A systematic review suggests that two to three safety incidents occur in every 100 consultations; 4% of these were associated with severe harm (Panesar et al., 2016).

Safety huddles, ‘brief, daily, focused team meetings involving all professional and clinical managerial staff in a non-judgmental setting’ (Cracknell, 2017), are a well-established concept in high reliability organisations (Goldenhar et al., 2013). A systematic review of safety briefings in various healthcare settings suggests positive outcomes after safety huddle introduction, including improved risk identification, enhanced relationships, increased incident reporting and ability to voice concerns (Ryan et al., 2019). In the United Kingdom, safety huddles have been introduced into secondary care with some promising early evaluation (Cracknell et al., 2016). However, the feasibility and acceptability of similar meetings in primary care are unknown and there have been no formal evaluations. We therefore explored the feasibility and acceptability of huddles in general practice.

## Methods

### Survey

We adapted an existing questionnaire on patient safety huddles (Alison Lovatt, personal communication, HUSH), which was based on the Theoretical Domains Framework (Michie et al., 2005) and the Improvement Academy’s Achieving Behaviour Change Toolkit (Yorkshire Quality and Safety Research Group, 2017), to use in general practice and refined questions on a convenience sample of general practitioners (GPs). We distributed the questionnaire electronically to all 314 practices in West Yorkshire (Supplementary Material, Appendix 1). We assessed findings using descriptive statistics.

### Interviews

We sought interest from primary care professionals attending postgraduate meetings in Leeds during early 2018. We conducted semi-structured interviews based on the survey findings and exploring work schedules, meeting frequency and content, and individual’s experiences and views of safety huddles. We attempted to include a reasonably diverse sample of clinical and non-clinical staff from multiple practices. We initially planned to recruit a sample of around 20 participants and then judge the degree to which data saturation had been achieved.

We obtained written, informed consent prior to all interviews, which were recorded on an encrypted electronic device and transcribed *verbatim*. We used a framework approach to analysis. As the interviews were partially dictated by the findings of the survey, our *a priori* knowledge shaped the framework. One researcher (H.P.) familiarised herself with the transcripts, identifying issues and emerging concepts; sections of text were then coded, indexed and then charted into the framework. The coded concepts were subsequently interpreted (Srivastava and Thomson, 2009).

## Results

In total, 34 general practices (practice response rate 11.1%) completed our survey, including 47 individuals participated with varying practice roles, duration of experience and sex (Table 1).

**Table 1.** Characteristics of survey respondents

Characteristics	Number (%)
Job role	
GP partner	13 (27.7)
Salaried/locum GP	6 (12.5)
Practice manager	14 (29.8)
Nurses	7 (14.6)
Pharmacists	2 (4.2)
Research and administration staff	2 (4.2)
Foundation doctors	3 (6.3)
Experience	
<5 years	11 (23.4)
5–10 years	9 (19.1)
10–20 years	13 (27.7)
>20 years	14 (29.8)
Sex	
Female	32 (68.1)
CCG	
All Leeds	23 (48.9)
Others in West Yorkshire	24 (51.1)

CCG – Clinical Commissioning Group.

Twenty-two practices (64.7%) reported meeting for staff breaks, whilst eight (23.5%) reported that such meetings had ceased (Table 2).

**Table 2.** Summary of survey and interview findings

Theme	Survey statements and interview questions	Survey response by practice^a^ (%)	Survey response by individual (%)	Interview quotations
Time	We have not got time for huddles	Strongly agree/agree 14 (44.7%)	Strongly agree/agree 21 (44.6%)	‘I think the hardest thing would be finding the time to do it. That would be the biggest constraint’. ‘The main concern will be have I the time? We have many extra patients, the admin time gets done after you are supposed to finish’. ‘You’re not going to prioritise safety huddles…if you’ve got like 4 patients waiting’.
Meeting frequency	Do you have breaks between surgeries where staff might meet for coffee for a few minutes?	Yes, 22 (64.7%)	Yes, 27 (57.5%)	‘Some of us have a kind of break at 10 o’clock in theory. But… we’re not getting it. Or run out at different times in that half hour to grab our tea’ ‘We’ll have 2 appointments in the morning blocked out. But they’re at different times so I don’t get to see my colleagues’. ‘I met very briefly with another GP this morning and I felt guilty because there was another doctor slogging away whilst we were having coffee’. ‘[Breaks are] becoming less and less achievable’.
	Do you have regular meetings?	Yes, 17 (50%)^b^	Yes, 24 (51.1%)^b^	‘We do have regular meetings perhaps every 2 to 3 weeks’. ‘There has been a limitation on the some of the meetings… we manage one every 3 or 4 weeks’. ‘We have a full practice meeting twice a year’.
Inclusivity	It is not possible to get people together so we can huddle	Strong agree/agree 14 (44.7%)	Strongly agree/agree 18 (38.3%)	‘There isn’t a natural time when you would happen to all be together’. ‘Its nice theory to have a meeting but we need to use the workload of general practice’.
	Who attends the meetings?	–	–	‘The part-time staff they can usually come to one a fortnight’. ‘I don’t attend the weekly meeting…it suits everybody else to have them first thing on a morning and it doesn’t suit me’. ‘It doesn’t include non-clinical staff…there’s no meetings that include everybody’. ‘Not everybody gets to go. It depends on whether they work that day’.
Current culture	Staff at our practice are keen about holding huddles	Strongly agree/agree 9 (26.5%)	Strongly agree/agree 11 (23.9%)	‘Ideas like safety huddles…people like GPs are sick of mandatory changes’. ‘Just because it’s a good idea in secondary care does not make it a good idea in primary care’. ‘I can think of one doctor who wouldn’t come… some would, you know, need a bit of em… persuasion’. ‘Like the culture of very much that we’re busy and you’ve got to get your head down and crack on’.
	Huddles will reduce harm to patients	Strongly agree/agree 22 (64.7%)	Strongly agree/agree 31 (65.9%)	‘You can run certain patients past each other’. ‘It’s a convenient time to get opinions from other people’. ‘I think they’re essential’ ‘I think [safety huddles] sound like a really good idea, you know, getting together…safety netting and team working’.
Team relationships	(Theme emerged from analysis of interviews)	–	–	‘If you spend time together you can empathise more…Getting to know each other better. Getting to trust each other more’. ‘[It] is an opportunity to build a relationship [with non-clinical staff] and keep morale up’. ‘Another day and you haven’t seen half your colleagues! I don’t necessarily know each other’. ‘If there’s been a lot of staffing changes and upheaval you need to sort of…consolidate and em engender a bit of team spirit’.

a Counted as ‘yes’ for whole practice when over half of individuals from that practice responded yes.

b Includes daily, weekly and monthly meetings.

Lack of time was identified as the main barrier to adopting huddles and less than a third of respondents agreed they would be keen to start safety huddles (9; 26.5%).

In terms of facilitating factors more than half of the staff felt huddles may reduce harm (31; 65.9%); the majority of respondents felt they had the communication skills needed to contribute to huddles (41; 87.3%) and suitable places within the practice to huddle (32; 68.0%).

Seven professionals participated in interviews. Five key themes were identified: time, meeting frequency, meeting inclusivity, staff culture and relationships (Supplementary Material, Appendix 2).

*Time.* Consistent with survey findings, lack of time was the biggest barrier to either taking breaks or introducing safety huddles. Interviewees believed that time for breaks or meetings might be taken out of available appointments, meaning fewer patients would be seen or that clinics would overrun. There was an expectation that huddles or breaks would not be a priority in a busy system.

*Meeting frequency.* This varied, although no practice met more frequent than weekly. A variable staff working pattern, for example, working less than full-time, further hindered timetabling of meetings. Often this was tackled by meetings being on alternating days; however, this meant certain individuals were only able to attend a maximum of half all scheduled meetings and some could not attend any. Although some practices had a scheduled break, interviewees reported that, in reality, none of them managed this.

*Inclusivity.* Most practices had multiple meetings for different staff groups, for example, partners meetings, clinical meetings and practice meetings. Frequently, these meetings only included the clinical staff; others might include the practice manager but rarely other non-clinical staff such as receptionists. As with meeting frequency, less than full-time working patterns often prevented individuals from attending meetings.

*Current culture.* General practice was perceived by interviewees as a relatively isolating profession, with individuals working independently in separate rooms; this contrasts hospitals where multidisciplinary teams, physically working together, are the norm. Interviewees used this independent culture as an argument both for and against meetings and huddles. Interviewees believed introduction of huddles would be resisted due to the perception of them as a secondary care intervention, which would not fit with primary care working culture and structure. However, some individuals expected that meetings could provide clinical support within growing culture of GPs working in isolation.

*Team relationships.* Meetings and breaks can nurture team relationships and reduce conflict, particularly between individuals who do not work closely together. Scheduled meetings or breaks were perceived as important for newer employees and as clinical support networks. Interviewees also revealed the experience of guilt on casual interactions with colleagues, such as having coffee outside of allocated times. This is due to the experience that other colleagues may be unable to take a break that day and is closely related to the perception of workload, scheduling and time efficiency.

## Discussion

Patient safety huddles appear promising in secondary care, but there are distinct challenges to their introduction in primary care. We identified time as the greatest barrier, along with workload pressures, and uncertainty about who should participate. Practice staff now appear to have fewer opportunities for meeting, but did recognise the potential professional and social value of huddles.

We believe that our modest, exploratory study is the first to assess the feasibility of huddles in UK primary care. However, our survey has not been validated for use in general practice and we only had limited time available for the interview study and recognise that we did not achieve data saturation. Our findings are susceptible to social desirability and respondent bias; we attempted to minimise the former by making the survey anonymous.

O’Malley *et al.* reported positive experiences in a small US primary care study, where 23 of 27 practices using huddles regularly perceived their value in enhancing staff communication (O’Malley et al., 2015). Our interviewees generally considered that safety huddles were challenging to integrate within existing primary care time and resources.

Riley *et al.* identified that in a field where levels of stress and burnout are high, culture change and access to support are crucial to enable GPs to do their job effectively (Riley et al., 2018). Currently, new opportunities for practices to meet are declining, which may have a perceived negative impact on practice relationships and contribute further to staff burnout (Hall et al., 2019).

General practice meetings are variable in terms of frequency and inclusivity. Overall, although staff are open to the idea of a huddle-type meeting, it is evident that the perceived positive benefits of team meetings are largely outweighed by the potential negative impact on other responsibilities. Given the small number of respondents in our study, we are cautious about its overall generalisability to UK general practice.

Any efforts to introduce huddles into general practice should consider tight time constraints and impact on staff morale, as well as sustainability. With growing evidence of individual pressure and burnout in GP (Riley et al., 2018; Hall et al., 2019), further research into the role and feasibility of safety huddles is merited. Changing ways of working within general practices during the COVID-19 pandemic may add impetus to such work (Thornton, 2020; O’Dowd, 2020). However, the key litmus test will ultimately be whether huddles can be shown to improve patient outcomes, specifically the reduction of safety incidents in general practice.

## Supplementary material

For supplementary material accompanying this paper visit http://dx.doi.org/10.1017/S1463423620000298. click here to view supplementary material
