# Supplementary material for: Exploring the feasibility of patient safety huddles in general practice
Source: Prim Health Care Res Dev. 2020 Jul 27;21:e24. doi: 10.1017/S1463423620000298 (PMC7443801; doi:10.1017/S1463423620000298)
Supplement: Supplementary file 1 [file S1463423620000298sup001.docx]

| Please complete the questions below by simply placing an ‘X’ the box (from strongly agree to strongly disagree). | | **Strongly agree** | **Agree** | **Neither** | **Disagree** | **Strongly disagree** |
| --- | --- | --- | --- | --- | --- | --- |
| 1 | I don’t know what content we would discuss in our huddles | 1 | 2 | 3 | 4 | 5 |
| 2 | A huddle is no different to other things we already do (e.g. practice team meetings, briefings, informal/ad hoc discussions) | 1 | 2 | 3 | 4 | 5 |
| 3 | I don’t know what is expected of me in huddles | 1 | 2 | 3 | 4 | 5 |
| 4 | Huddles are just another initiative | 1 | 2 | 3 | 4 | 5 |
| 5 | My role means my views are (would be) less important than other members of staff attending a huddle | 1 | 2 | 3 | 4 | 5 |
| 6 | It is not my job to express concerns about patients in a huddle | 1 | 2 | 3 | 4 | 5 |
| 7 | There are no resources to address any problems we identify/discuss in the huddle | 1 | 2 | 3 | 4 | 5 |
| 8 | Huddles are only for medically trained staff | 1 | 2 | 3 | 4 | 5 |
| 9 | I am confident in attending/leading a huddle | 1 | 2 | 3 | 4 | 5 |
| 10 | I don’t have the communication skills to contribute to huddles | 1 | 2 | 3 | 4 | 5 |
| 11 | There is nothing more we can do to reduce harm | 1 | 2 | 3 | 4 | 5 |
| 12 | Huddles will reduce harm to patients | 1 | 2 | 3 | 4 | 5 |
| 13 | There are no benefits to me/other staff in attending huddles | 1 | 2 | 3 | 4 | 5 |
| 14 | I am worried I may appear incompetent/lack knowledge about my patients if I (were to) contribute to a huddle | 1 | 2 | 3 | 4 | 5 |
| 15 | We haven’t got time for huddles | 1 | 2 | 3 | 4 | 5 |
| 16 | Huddles (would) take staff away from important care duties | 1 | 2 | 3 | 4 | 5 |
| 17 | There are more important things I need to do other than attending huddles | 1 | 2 | 3 | 4 | 5 |
| 18 | I am worried I may say something inappropriate when I contribute in huddles | 1 | 2 | 3 | 4 | 5 |
| 19 | I(d) forget to attend huddles | 1 | 2 | 3 | 4 | 5 |
| 20 | It is part of my routine to attend huddles | 1 | 2 | 3 | 4 | 5 |
| 21 | I (would) feel uncomfortable about contributing to huddles | 1 | 2 | 3 | 4 | 5 |
| 22 | I am the only one (or one of only a few people) who (are likely to) contribute to our huddle | 1 | 2 | 3 | 4 | 5 |
| 23 | Patient turnover is too quick to make a huddle worthwhile | 1 | 2 | 3 | 4 | 5 |
| 24 | It is not possible to get people together so we can huddle | 1 | 2 | 3 | 4 | 5 |
| 25 | Staff at our practice are keen about holding huddles | 1 | 2 | 3 | 4 | 5 |
| 26 | There is nowhere suitable to huddle | 1 | 2 | 3 | 4 | 5 |

**Appendix 1**

**Please contact the Authors Directly for the full survey**

**Appendix 2**

| **Themes** | **Categories** | **Codes** | **Concepts** |
| --- | --- | --- | --- |
| Time | Time | Efficiency | Appointments overrunning  Leaving work late/starting early |
|  |  | Workload | Home visits  Referrals  Administrative tasks  Additional tasks |
| Meeting frequency | Meetings | Informal/formal meeting  Clinical/non-clinical meeting | Multiple meetings of different staff  Different types of meetings |
|  | Breaks | Coffee breaks | Natural times in the day to meet |
| Inclusivity | Teams | Separate teams | Clinical vs non-clinical team  Doctor team  Nursing team  Partners  Salaried GPs  Locum GPs |
|  | Schedules | Working hours  Working pattern | Less than full time staff  On-call duties |
| Culture | Structure | Isolation  Independent working | Separate rooms to work in  Seeing patients individually  Making decisions independently  Individual responsibility |
|  | Primary vs Secondary care | Different to hospitals  Differences between practices | MDT/ team discussion of patients  Daily roles and responsibilities |
| Relationships | Communication | Communication  Professional guidance  Value of staff | Ad hoc clinical guidance  Unclear roles/relationships  Unclear contributions |
|  | Support | Personal support  Grievances  Trust | Not knowing colleagues  Lack of socialising in/out of work |

**Appendix 2:** Table demonstrating the framework used for qualitative analysis of interviews
